# Effect of vitamin B complex administration on pain and sensory problems related to inferior alveolar nerve damage following mandibular implant placement surgery

**DOI:** 10.34172/japid.2022.007

**Published:** 2022-05-30

**Authors:** Shima Ghasemi, Amirreza Babaloo, Mehrnoosh Sadighi, Zeinab Torab, Hamidreza Mohammadi, Elshan Khodadust

**Affiliations:** ^1^Department of Prosthodontics, Faculty of Dentistry, Tabriz University of Medical Sciences, Tabriz, Iran; ^2^Department of Periodontics, Faculty of Dentistry, Tabriz University of Medical Sciences, Tabriz, Iran; ^3^Department of Pediatric Dentistry, Faculty of Dentistry, Tabriz University of Medical Sciences, Tabriz, Iran; ^4^Private Practic, Isfahan, Iran

**Keywords:** Dental implants, Nerve injury, vitamin B complex

## Abstract

**Background:**

Inferior alveolar nerve damage can lead to mild to severe paresthesia and even facial pain. One of the treatments considered today for the reconstruction and treatment of damaged peripheral nerves is the use of vitamin supplements. This study aimed to evaluate the effect of vitamin B complex supplementation on pain and sensory problems following mandibular implant placement surgery.

**Methods:**

In this single-blind clinical trial, 46 patients applying for implant placement, who were eligible for the study, were evaluated. All the patients were examined for sensory facial injury and inferior alveolar nerve injury within 24 hours after implant placement. The nerve damage was recorded by AI (asymmetry index) in the initial examination. Patients who reported clinical and radiographic signs of nerve damage due to implant or drill impingement of the nerve canal were excluded from the study and promptly treated with anti-inflammatory protocols. Then the patients were randomly divided into control (n=23) and intervention (n=23) groups. Patients in the control group received routine treatment after implantation, and patients in the intervention group received vitamin B complex pills in addition to routine treatment. A placebo was used to eliminate the inductive effect of the drug in the control group. Follow-up of patients was performed at intervals of 14 days and 1, 2, and 3 months after treatment. Data analysis was performed using SPSS 24 and Kruskal-Wallis, Wilcoxon, and chi-squared tests.

**Results:**

In both groups, a decreasing trend in pain intensity was observed for up to three months. There were no significant differences between the mean pain intensity in the intervention and control groups at all the follow-up intervals. In both groups, a decrease in the rate of paresthesia was observed during the 3-month follow-up. The mean of paresthesia in the two groups was not significantly different at any follow-up interval.

**Conclusion:**

Vitamin B complex might not affect pain intensity and paresthesia after implant surgery.

## Introduction

 Today, the number of people undergoing implant surgeries has increased significantly compared to the last 15 years. Also, now that the level of confidence of surgeons has increased, they are working on more complex cases, which can lead to an increase in problems and complications.^1^One possible complication following mandibular implants is damage to the trigeminal nerve branches (including the inferior alveolar, lingual, mental, and infraorbital nerves). Nerve damage can generally occur following direct trauma, inflammation, or infection.^2^ Injury can occur through the following processes: anesthesia, flap reflection, osteoplasty, osteotomy, and implant placement. Because healing a damaged nerve is associated with many issues, it is necessary to prevent nerve damage. Therefore, sufficient knowledge is necessary about the anatomy and histology of oral nerves, clinical signs and symptoms of patients with nerve damage, and methods of diagnosing neuropathies of these nerves. Before diagnosing nerve damage, therapeutic goals should be adopted regarding the examination and registration of the patient’s clinical symptoms, drug treatments, implant removal, and patient referral or a combination of the above methods.^3-5^

 The nervous system can be generally divided into two parts: central and peripheral.^6^ The peripheral nervous system includes the cranial and the spinal nerves, with axons and dendrites. Peripheral nerves can also be sensory, motor, or sensory-motor.^7^The trigeminal nerve is the fifth and largest cranial nerve.^8^ This nerve consists of three main branches: ophthalmic (V1), maxillary (V2), and mandibular (V3). The mandibular branch is the largest and innervates the lips, chin, teeth, surrounding soft tissues, mandible, and external ear. Unlike sensory fibers, motor fibers of the mandibular nerve are not usually damaged during implant placement surgery because they separate from the V3 nerve before leaving the oval cavity.^9^ Following implant placement, an incidence of 0‒36% for permanent nerve damage has been reported in various studies, leading to sensory impairment in the lips.^10-12^ Experimentally, many nerve injuries occur when vestibular incisions are made for better accessibility and visibility. Today, CT-scan imaging has significantly reduced this kind of injury. In this case, recent studies have reported much lower rates of nerve damage than previous studies. A studyreported that transient trigeminal nerve damage following dental implant placement was 2.95% (5 out of 169 patients).^13^ Recent studies have reported that damage to the inferior alveolar nerve following implant placement might occur in 0‒40% of cases.^14,15^ This injury is one of the unpleasant experiences ranging from mild to complete numbness and even facial pain.^16^ Following this injury, many human functions, such as talking, eating, putting on makeup, drinking, and shaving the face, may be affected.^17^

 Remedies for peripheral nerve damage include pharmacotherapy (including systemic steroids, gabapentin, and carbamazepine) and surgical interventions.^18^

 Vitamin B is commonly used to treat peripheral neuropathy, but it is not clear if it is useful. This review of 13 trials on diabetic and alcoholic peripheral neuropathy with 741 participants showed only one study that suggested possible short-term benefits from eight-week treatment with benfotiamine (a derivative of vitamin B_1_) with slightly greater improvement in vibration perception threshold compared to placebo. When given in a higher dose for four weeks, vitamin B complex was more efficacious than a lower dose in reducing pain and other clinical problems based on another study. Two to eight weeks of treatment with vitamin B was less efficacious than alpha-lipoic acid, cilostazol, or cytidine triphosphate in a short-term improvement of clinical and nerve test findings. All these findings require confirmation in larger studies before they can be accepted as definite. Vitamin B is generally well-tolerated, with only a few reports of mild side effects.^19^

 One of the treatments considered today for the regeneration and treatment of damaged peripheral nerves is the use of vitamin supplements. Recent studies have shown that vitamins such as vitamins D, C, and B can accelerate the regeneration of peripheral nerves.^19^ Among them, it has been observed that B vitamins, especially vitamins B_1_, B_6_, and B_12_, are the most important therapeutic factors in accelerating the regeneration of damaged peripheral nerves.^20^

 Fernández-Villa et al^21^ showed that B vitamins, especially in combination with folic acid, can significantly accelerate the healing process of the damage, leading to peripheral nerve dysfunction.

 A meta-analysis evaluated the efficacy and safety of mecobalamin tablets in peripheral neuropathy. Mecobalamin is an active form of vitamin B_12_ useful in improving nerve regeneration and neuropathic pain symptoms. It is widely used in Asia to treat peripheral neuropathies, but its effect is still unclear. This review evaluated the effectiveness of clinical therapy, pain scores, neuropathic symptom scores, nerve conduction velocity (NCV), and mecobalamin’s side effects. Mecobalamin was evaluated alone and in combination. For NCV results, only combination therapy with mecobalamin was effective. Mecobalamin alone and in combination did not affect pain score and neuropathic symptom outcomes. No serious side effects have been reported with this product during treatment. The findings suggest that mecobalamin in combination may effectively improve the therapeutic effect and outcomes of NCV in patients with peripheral neuropathy, but the evidence for mecobalamin is unclear. Further high-quality studies are required to confirm this finding.^22^

 It is noteworthy that inferior alveolar nerve damage is very common and averaged 64.4% among all trigeminal nerve injury referrals. Damage to this nerve affects the patient’s quality of life, and the iatrogenic nature of this injury also leads to negative psychological effects.^3^ When this injury occurs, the dentist is required to care for and treat the patient as soon as possible. On the other hand, due to recent vitamin-based therapies, many studies have been performed to evaluate the therapeutic role of B vitamins alone or in combination with other vitamins in the regeneration of damaged peripheral nerves. Therefore, this study aimed to investigate the effect of vitamin B complex supplementation on pain and sensory problems following implant placement surgery in the posterior mandible.

## Methods

###  Study design 

 In this single-blind randomized clinical trial, with the ethical code IR.TBZMED.REC.1399.551, patients were evaluated after meeting the inclusion criteria. The study began after the approval of the Ethics Committee of Tabriz University of Medical Sciences.

###  Study population

 Healthy patients who needed dental implants in the posterior mandibular area participated in this study. The patients were informed of their role in the study, possible postoperative complications, and the advantages and disadvantages of surgical procedures. They signed an informed consent form to participate in the study.

###  Exclusion criteria

 The exclusion criteria included the following:

1. Patients needing extraction and immediate implant placement
2. Smokers
3. Patients with medical problems such as diabetes, osteoporosis, blood dyscrasias, and malignancies
4. Patients with poor oral hygiene
5. Patients with altered sensation in the lower lip due to previous mandibular surgery or third molar extraction
6. Patients with untreated periodontal disease.

###  Study protocol

 All the patients were examined for sensory facial damage and inferior alveolar nerve damage within 24 hours after implant placement. Nerve damage was performed by the IANIDIS protocol and by the project supervisor. Information about nerve damage, including etiologic factors of nerve damage (during and after surgery) and injury mechanisms, was recorded.

 Patient information, including age, gender, the position involved, number of teeth operated on, and systemic disease, were recorded.

 The severity of nerve damage was recorded by the pinprick test^23^ in the initial examination. Patients reporting nerve damage symptoms and clinical and radiographic signs of nerve damage due to implant or instrument impingement on the nerve canal were excluded from the study and promptly treated with anti-inflammatory protocols (corticosteroids, NSAIDs, etc.)

 Randomization was performed by placing 23 red (controls) and 23 green (test) marbles in a bag, from which the patient selected one.

 The patients were given 1 g of amoxicillin one hour before surgery and 500 mg every eight hours for one week. Treatment was carried out under local anesthesia with local infiltration buccally and lingually. A crestal incision was made in the surgical area, and divergent releasing incisions remote to the defect area were used if needed. A full-thickness flap was elevated. The proposed implant site was prepared according to the manufacturer’s recommendation. The implant with the desired length was placed in an ideal position about 3 mm apical to the cementoenamel junction of the adjacent teeth. Guided bone regeneration was used if needed (mixture of bovine bone [particle size, 0.25–1.0 mm] and CaSO_4_ [ratio, 4: 1], covered with a layer of CaSO_4_). The flap was secured using 4-0 Vicryl interrupted sutures. The patients were given NSAIDs (ibuprofen, 600 mg) and chlorhexidine mouthwash for one week after surgery.

 The patients in the control group received routine treatment after implantation. In addition to routine treatment, patients in the intervention group received daily vitamin B complex tablets, including 5 mg of vitamin B_1_, 2 mg of vitamins B_2_ and B_6_, and 20 mg of nicotinamide starting immediately after implantation. A placebo was used to eliminate the inductive effect of the drug in the control group. Vitamin B complex was administered as an adjunct and with the patient’s consent and was not part of the standard treatment protocol.

 Patients were given a questionnaire to evaluate pain during anesthesia and implant surgery. The severity of pain was assessed using a 10-cm visual analog scale, labeled as “no pain” at the zero extreme and “severe pain” at the 10-cm mark. Changes in sensation in the field were evaluated by standard neurosensory examination tests^23^ at intervals of 14 days and 1, 2, and 3 months after treatment.

**Figure 1:**
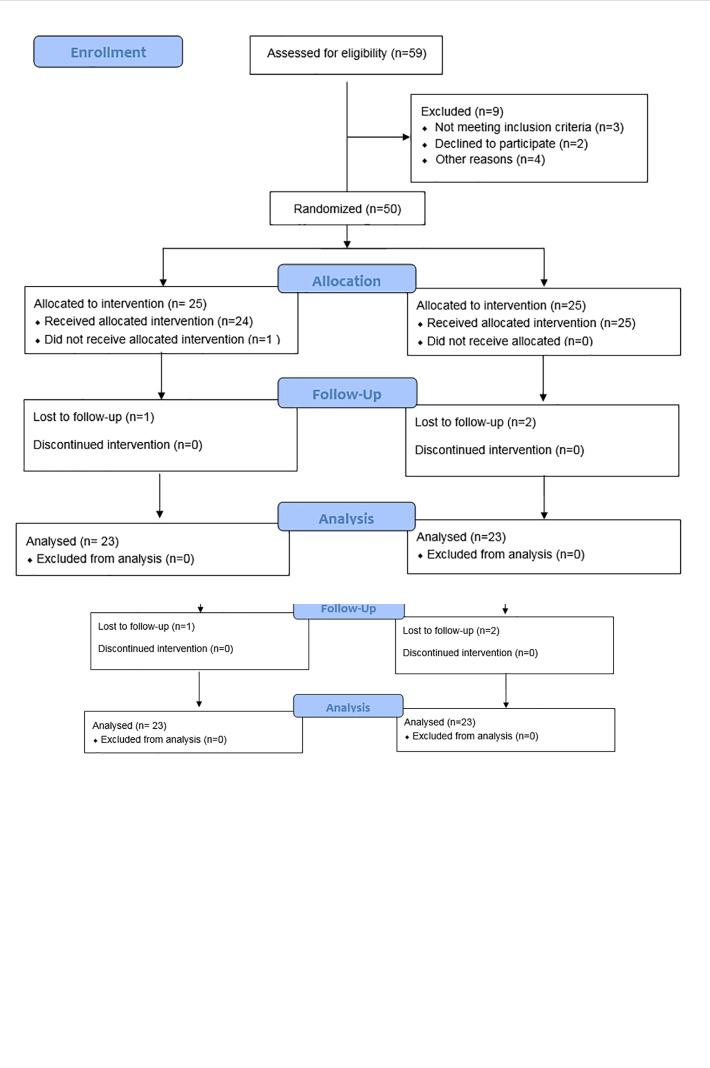
Consolidated Standards of Reporting Trials Diagram

 To improve the reporting of RCT, we followed the Consolidated Standards of Reporting Trials Diagram (Figure 1).

###  Data analysis

 The data were reported using descriptive methods (frequencies and percentages). Kruskal-Wallis and Wilcoxon tests were used to assess the severity of pain, and the chi-squared test was used to assess sensory symptoms. Data analysis was performed using SPSS 24. A probability of <5% was considered a significant level.

## Results

 In this study, 23 patients in the control group and 23 in the intervention group were studied. In the control group, 43.5% of the patients were male, and 56.5% were female. In the intervention group, 39.1% were male, and 60.9% were male.

 According to Table 1, a review of pain intensity showed that at the 14-day interval, the pain intensity in the control group was not significantly different from the intervention group (P=0.092). After one month, the pain intensity in the control group with was not significantly different from the intervention group (P=0.120). At two months, the pain intensity in the control group was not significantly different from the intervention group (P=0.068). At the 3-month interval, the pain intensity in the control group was not significantly different from the intervention group (P=0.206).

**Table 1:** Comparison of pain intensity between the two groups at all the follow-up intervals

**Time**	**Control group (n=23)**		**Intervention group (n=23)**		P-value*
	**Mean**	**SD**	**Mean**	**SD**	
14 days	5.22	1.00	4.65	0.98	0.092
30 days	3.04	1.02	2.30	1.61	0.120
60 days	1.87	1.91	0.96	1.69	0.068
90 days	0.87	1.71	0.04	1.36	0.206

*P-value: Mann-Whitney U test

 According to Table 2, the evaluation of paresthesia showed that at the 14-day interval, the rate of paresthesia in the control group did not differ significantly from the intervention group (P=0.052). At the 1-, 2-, and 3-month intervals, the rate of paresthesia in the control group did not differ significantly from the intervention group (P=0.690, P=0.681, and P=0.771, respectively).

**Table 2:** Comparison of the rate of paresthesia between the two groups at all the follow-up intervals

**Time**	**Control group (n=23)**		**Intervention group (n=23)**		P-value*
	**Mean**	**SD**	**Mean**	**SD**	
14 days	4.70	0.93	4.04	1.26	0.052
30 days	2.65	1.75	2.91	1.50	0.590
60 days	1.83	1.37	2.00	1.48	0.681
90 days	1.13	1.01	1.22	1.00	0.771

*P-value: Mann-Whitney U test

## Discussion

 The inferior alveolar nerve (IAN) carries the general sensation for the mouth, teeth, lips, and chin. It serves important functions for oral health and general functions such as eating, chewing, tasting, and phonation. Inferior alveolar nerve damage can occur during various dental surgical procedures (wisdom tooth extraction, reconstructive surgery on the lower jaw, orthognathic surgery, surgical removal of cysts, inferior alveolar nerve lateralization, and dental implant placement). The development of these complications can be an important factor in reducing the quality of life. One of the problems of dental implantology is the prevention of complications. During implant insertion, different inferior alveolar nerve disorders are caused by direct perforation of the inferior alveolar nerve canal, intraosseous hematoma, or nerve pressure with an implant. Due to hematoma, minor injuries to the IAN may develop, edema may develop later, and the injury may worsen.

 In cases where the patient reports permanent paresthesia on the side of implant placement, including the lower lip and chin (3 hours after surgery when the effect of local anesthesia wears away), this will be the first sign of nerve damage. Patients with post-traumatic trigeminal neuropathy can exhibit neuropathic pain, anesthesia, hypesthesia, or hyperesthesia, leading to stress disorders. Many tools are available for assessing nerve injury severity. Clinical neurosensory tests (pinprick sensation and thermal sensation) are the most commonly reported diagnostic tests undertaken. Any damage (penetration or compression) and hemorrhage in the mandibular canal lead to intraoperative pain of the neuralgic type. Ischemia itself, even without direct damage to the nerve, will cause sufficient inflammation and damage to the nerve, possibly leading to permanent damage to the nerve. The degree of pain relief is individual and depends on each patient. The issue of regeneration of nerve tissue and pathomorphological changes in the nerve fiber when it is damaged during dental implantation is discussed in research publications.^5,12,17^ The variety of proposed treatment methods and the lack of universal tactics indicate the need to develop new methods to treat post-traumatic neuropathy of the inferior alveolar nerve. The conservative therapies for inferior alveolar nerve neuropathy include corticosteroids and non-steroidal anti-inflammatory agents.^23^ In the absence of effective methods to resolve post-traumatic neuropathy of the inferior alveolar nerve, alternative treatment methods, including physiotherapy, have been recommended.

 In the present study, the mean pain in the two groups had a decreasing trend in pain intensity for up to three months. This decreasing trend was slightly higher in the intervention group, but there was no statistically significant difference between the two groups.

 A study on the effect of B vitamins on reducing pain in patients with chronic mechanical back pain showed that vitamins B_1_ and B_6_ had no effect on improving chronic mechanical low back pain, and currently, the use of these vitamins is recommended to physicians based on clinical experience,^24^ consistent with the present study.

 Sawangjit et al^21^ also found in a meta-analysis that mecobalamin, an active form of vitamin B_12_, was useful in improving nerve regeneration and neuropathic pain symptoms, although more high-quality studies were needed.

 There are several risk factors for developing persistent pain after surgery. However, the prevalence of sensory and neurologic defects after dental implant placement is relatively low. Many factors may contribute to a neurological defect, including changes in implant techniques, the operator’s skill, proximity to the nerve canal, and even the patient’s physiological condition.^25-29^

 Buesing et al^30^showed in a systematic review that vitamin B_12_ might be an adjunctive or integrated treatment for pain. The researchers concluded that vitamin B_12_ and other painkillers, including non-steroidal anti-inflammatory drugs, could reduce pain and neuralgia; however, larger, double-blind, placebo-controlled trials are required for definitive results.

 Zhang et al^31^ showed that methylcobalamin (an activated form of vitamin B_12_) works as an adjunct to pain relief by protecting damaged nerves.

 In the present study, paresthesia in both groups had a decreasing trend for up to three months. This decreasing trend was slightly higher in the intervention group, but there was no statistically significant difference between the two groups.

 Recent studies have reported a damage rate of 0‒40% to the inferior alveolar nerve following implant placement.^32^

 Bartling et al^33^ studied the prevalence of sensation change in 94 patients after placing mandibular dental implants. The researchers found that 8.5% of patients reported a change in sensation during the first visit after implant placement. All the subjects reported complete remission of symptoms within four months (121 days). One person reported complete paresthesia for two months.

 Another study showed that following implant placement surgery, 31.25% of patients had hypersensitivity, and 68.75% of patients had hyposensitivity following inferior alveolar nerve damage.^34^

 Studies have also shown that paresthesia of the lips occurs after implant placement in 7‒10% of cases, and in 3% of patients, there were still sensory changes two years after implant placement.^35,36^

 In the present study, three months after implant placement, 30% of the patients in the intervention group and 39% in the control group had paresthesia with different intensities.

 Recent studies have reported inferior alveolar nerve injuries in 0‒40% of patients after implant placement.^14^

 This wide variability may be attributed to a range of factors, such as the variability of implant placement techniques, surgical skills, nerve canal proximity, the patient’s physiologic condition, and the lack of documentation on neurologic function evaluation.^33,37^

 In addition, published reports vary widely in terms used to describe patients’ symptoms after nerve injury. The term paresthesia was originally used to describe several forms of sensory changes reported by patients, including pain, warmth, coldness, burning sensation, numbness, and itching.

 Nerve pain or neuropathic pain is an unpleasant sensory experience often caused by damage to nerve cells.

 The difference between lower alveolar nerve damage and other types of sensory nerve damage is that these injuries are mostly iatrogenic and usually do not improve within eight weeks of surgery. Injuries very close to the nerve can also lead to delays in the diagnosis and treatment of patients.^2^ On the other hand, damage to this nerve affects the patient’s quality of life, and the iatrogenic nature of this injury also leads to negative psychologic effects.^3^

 Researchers introduced vitamin B_12_ as a treatment for peripheral neuropathic pain in a systematic review.^38^Fernández-Villa et al^20^ showed the accelerating role of B vitamins and folic acid in the regeneration of peripheral nerve damage.

 Improved treatment regimens are sought. One recommended treatment for neuropathic pain is vitamin B_12_, which is thought to relieve pain through several mechanisms, including enhancing myelination, increasing nerve regeneration, and accelerating the regeneration of peripheral nerve fibers.

## Conclusions

 In both groups, a decreasing trend in pain intensity was observed for up to three months. In addition, there was no significant difference between the mean pain intensity in the intervention and control groups at all the follow-up intervals. In both groups, a decreasing trend was observed in the rate of paresthesia for up to three months. At all the follow-up intervals, there was no significant difference in the mean of paresthesia between the intervention and control groups.

## Authors’ contributions

 HM initiated, conceptualized, and supervised the research work. HM, AB, and MS contributed to the design of the study. AB, HM, and MS performed the surgeries. ZT and EK performed data analysis and interpretation of data. ZT and HM, and AB wrote the manuscript. All the authors approved the final submission.

## Funding

 This work was supported by the Vice-Chancellor for Research, Faculty of Dentistry, Tabriz University of Medical Sciences, Tabriz, Iran.

## Availability of data

 The datasets used and/or analyzed during the current study are available from the corresponding author on reasonable request.

## Ethics approval

 This research was approved by the Research Ethics Committee of the Faculty of Dentistry under the code IR.TBZMED.REC.1399.551.

## Competing interests

 The authors declare no Competing interests related to the study.
